# Simultaneous transcatheter aortic valve implantation and femoral osteosynthesis: a case report

**DOI:** 10.1186/s40981-025-00769-w

**Published:** 2025-01-27

**Authors:** Yuki Mitsuta, Yukiko Okamura, Yosuke Miyamoto, Dai Tanahira

**Affiliations:** Department of Anesthesiology, Japanese Red Cross Kumamoto Hospital, 2-1-1, Nagamine-Minami, Higashi-Ku, Kumamoto, 861-8520 Japan

**Keywords:** Transcatheter aortic valve implantation, Aortic stenosis, Osteosynthesis, Hip fracture

## Abstract

**Background:**

Simultaneous cardiac and non-cardiac surgeries can be beneficial for patients, but there are still few reports on this approach.

**Case presentation:**

A 90-year-old woman was diagnosed with a femoral trochanteric fracture and severe aortic stenosis. A heart team conference decided to perform transcatheter aortic valve implantation (TAVI) and femoral osteosynthesis under general anesthesia on the same day. A preoperative simulation was conducted to review the surgical procedure and confirm the arrangement of the operating table and instruments. Preoperative management was carried out with attention to the risk of myocardial ischemia caused by bleeding or pain from the fracture. Transfemoral TAVI was completed without trouble, and after the patient was moved to the traction table, osteosynthesis was started. Back-up pacing with a temporary pacemaker was activated for atrioventricular block and bradycardia. After completing the surgery, the patient recovered from anesthesia, and extubation was performed after confirming the absence of paralysis. The patient had no noticeable postoperative complications and successfully underwent rehabilitation.

**Conclusions:**

Through meticulous preparation and perioperative management, we were able to perform TAVI and femoral osteosynthesis simultaneously, achieving a favorable outcome.

## Background

The number of patients with hip fractures has been increasing in Japan due to the progression of aging population, reaching 170,000 cases annually [1]. More than 2% of patients with hip fractures have severe aortic stenosis (AS) [2, 3]. It is recommended that surgery for hip fractures is performed within 48 h of injury [4]. However, the prognosis after femoral surgery worsens in patients with concomitant AS [3, 5]. Therefore, the decision to prioritize orthopedic surgery or aortic valve surgery must be made carefully [6]. However, there are few reports of performing cardiac and non-cardiac surgeries simultaneously.

Here, we report a case in which transcatheter aortic valve implantation (TAVI) and osteosynthesis were performed on the same day for a patient with a femoral trochanteric fracture complicated by severe AS.

## Case presentation

A 90-year-old woman (height, 147 cm; weight, 43 kg) was diagnosed with a right femoral trochanteric fracture after a fall. Preoperative transthoracic echocardiography revealed severe AS (aortic valve area, 0.7 cm^2^; maximum velocity, 4.1 m/s; and mean pressure gradient, 42 mmHg). Her medical history included hypertension, chronic heart failure, angina pectoris, and dementia. She was taking aspirin, antihypertensive drugs, and diuretics. Her electrocardiogram showed sinus rhythm with a heart rate of 63 bpm. Blood tests showed hemoglobin (Hb) level of 10.4 g/dL and brain natriuretic peptide level of 117 pg/mL. A heart team conference was held to determine the treatment strategy. The cardiologists proposed performing femoral osteosynthesis first, followed by TAVI, as they deemed the patient to have sufficient surgical tolerance. However, we anesthesiologists judged the perioperative cardiac risk to be high and suggested prioritizing the treatment of AS. After discussion, it was decided to perform transfemoral TAVI under general anesthesia first, followed by osteosynthesis on the same day.

Since this was our first case of simultaneous TAVI and non-cardiac surgery, as well as femoral surgery in a hybrid operating room, a simulation was performed by multiple professionals. The surgical procedure was reviewed, and the layout of the traction table for femoral surgery and instruments was determined with consideration of the base of the TAVI table, the surgical lights, and the X-ray equipment. Oral aspirin was discontinued upon admission, but Hb dropped to 9.7 g/dL in subsequent blood tests. Antihypertensive drugs were stopped due to a trend of decreasing blood pressure. Acetaminophen was regularly administered to manage pain from the fracture.

**Fig. 1:**
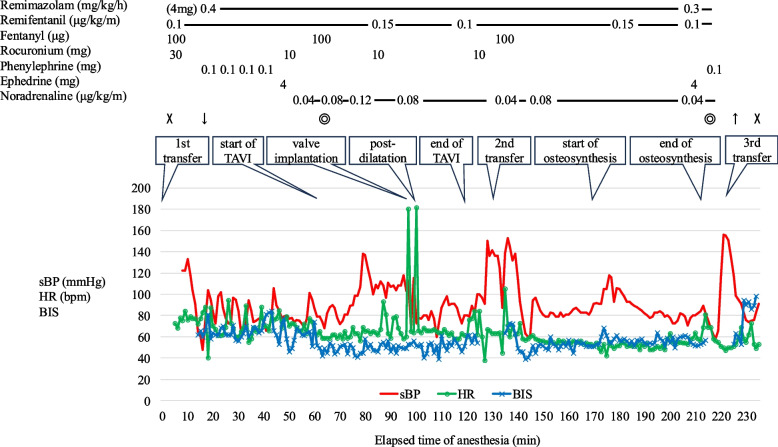
Anesthesia record. X, start and end of anesthesia; ◎, start and end of the operation; downward arrow, intubation; upward arrow, extubation; sBP, systolic blood pressure; HR, heart rate; BIS, bispectral index

**Fig. 2:**
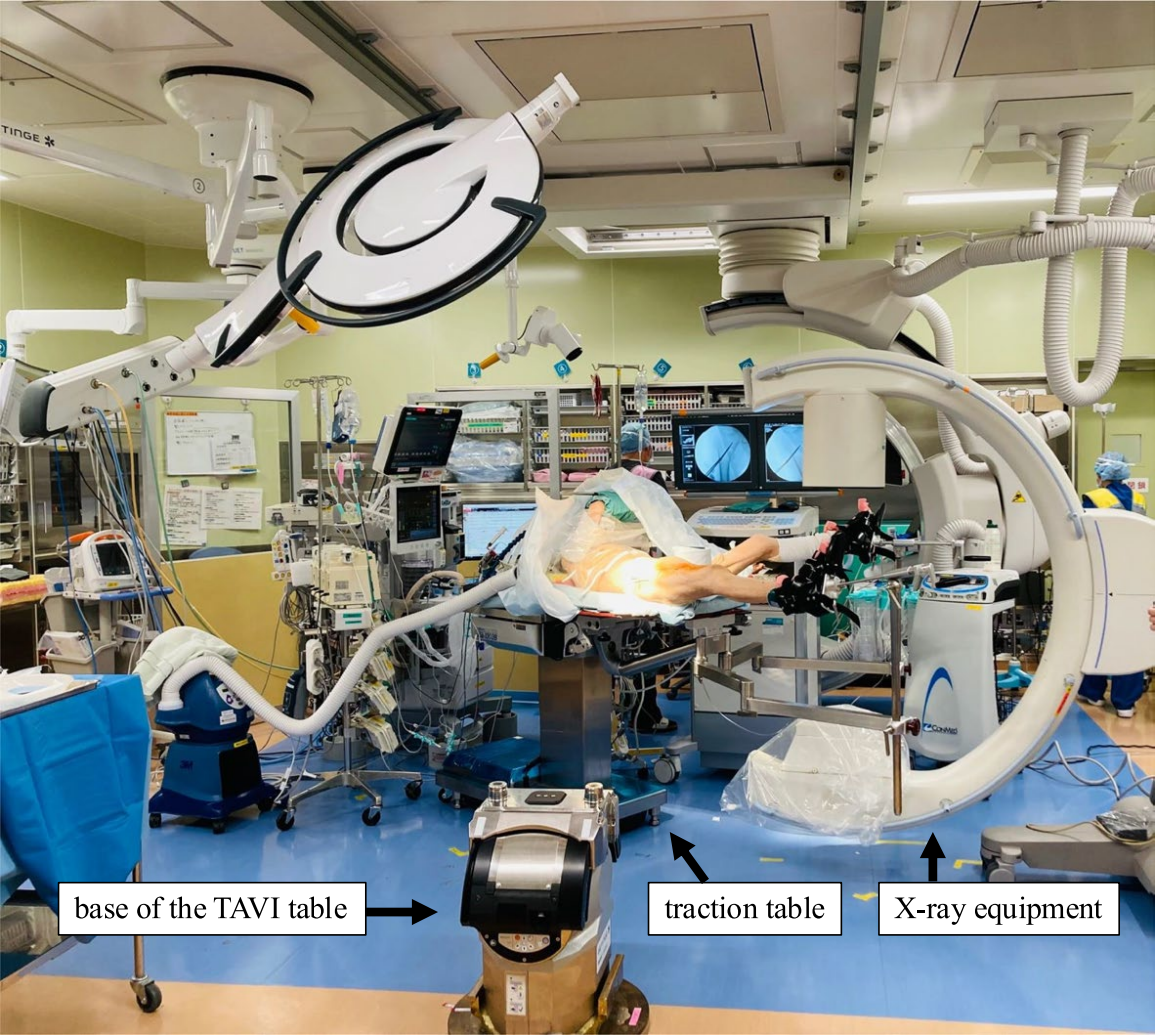
Femoral osteosynthesis in a hybrid operating room. The layout of the traction table and instruments was determined with consideration of the base of the TAVI table, the surgical lights, and the X-ray equipment

The patient was transported to the operating room on a stretcher, carefully transferred to the TAVI table, with attention to the fracture site. An arterial line was inserted into the left radial artery, and anesthesia was induced with 4 mg of remimazolam, 100 μg of fentanyl, and 30 mg of rocuronium, followed by endotracheal intubation (Fig. 1). A temporary pacemaker was placed through the right internal jugular vein. Anesthesia was maintained with remimazolam at 0.3 to 0.4 mg/kg/h, remifentanil at 0.1 to 0.15 μg/kg/m, and a total of 300 μg of fentanyl. Noradrenaline at 0.04 to 0.12 μg/kg/m was administered to maintain blood pressure. Heparin was administered to maintain activated clotting time of over 200 s. A balloon-expandable valve (SAPIEN 3™) was implanted under rapid pacing, and post-dilatation was also performed. Transesophageal echocardiography and angiography confirmed no abnormalities. Protamine was administered, and Perclose ProGlide™ was applied to the femoral artery, and TAVI was completed. Since blood pressure was maintained at 70% or more of the pre-anesthetic level with low-dose noradrenaline, we decided to proceed to orthopedic surgery. A compression band is usually attached to the groin for hemostasis after TAVI; however, it was not attached at this timing to avoid interference with the femoral osteosynthesis. The patient was moved from the TAVI table to the traction table and transitioned to osteosynthesis using an intramedullary nail (Fig. 2). First-degree atrioventricular block (AVB) and bradycardia were observed, and backup pacing was activated. As Hb dropped to 8.5 g/dL, 2 units of red blood cells were transfused. After osteosynthesis was completed, the patient was moved from the traction table to a ward bed, and a compression band was attached to the groin. 0.2 mg of flumazenil was administered, and the patient recovered quickly from anesthesia with no paralysis. She was extubated and transferred to the intensive care unit.

AVB and bradycardia gradually improved. Antiplatelet medication was started on the day after surgery. Rehabilitation began on postoperative day 2, and by day 11, the patient was able to walk with a walker. Without any significant complications, she was discharged from our hospital on postoperative day 17.

## Discussion

The patient was in a state of chronic heart failure due to severe AS and had been diagnosed with angina pectoris, with reduced exercise tolerance. According to Japanese guideline, the risk of perioperative cardiovascular complications would be high if femoral surgery were performed first, making it necessary to treat the AS beforehand [6]. Given the patient’s age, physical condition, and the urgency for femoral osteosynthesis, either balloon aortic valvuloplasty (BAV) or TAVI was considered as an option. BAV carries significant procedural risks with insufficient therapeutic benefits [7], so TAVI was chosen for AS treatment.

This case represents our first simultaneous performance of TAVI and non-cardiac surgery. One advantage of performing TAVI and femoral osteosynthesis simultaneously is the potential to shorten the waiting period for femoral surgery, which may lead to improved outcomes (Table 1). There are some reports of performing non-cardiac surgery a few days after TAVI, after confirming the absence of TAVI-related complications [8]. However, delaying surgery for hip fractures could worsen the patient’s prognosis [4]. By performing both TAVI and femoral surgery on the same day, the risk of perioperative cardiovascular complications can be reduced, while also shortening the waiting period, potentially leading to better outcomes.

**Table 1:** Advantages and disadvantages of simultaneous TAVI and femoral osteosynthesis

Advantages	• Femoral surgery can be performed early while reducing the risk of cardiovascular events associated with severe AS.
	• Shortening the waiting period for femoral surgery can improve prognosis.
	• Hemorrhagic and thrombotic complications may be reduced.
Disadvantages	• Emergency TAVI must be possible.
	• It is necessary to check the flow line and the arrangement of operating tables and instruments in the hybrid operating room.
	• Attention must be paid to post-TAVI complications such as complete AVB and stroke during femoral surgery.

The patient had been taking aspirin for angina, which was discontinued upon admission. Femoral trochanteric fractures typically result in an extracapsular hemorrhage of about 500 to 1000 mL. There are reports suggesting that antiplatelet therapy increases perioperative bleeding in patients with hip fractures, while other reports indicate that it does not increase bleeding [9, 10]. However, early surgery for hip fracture patients on antiplatelet drugs is associated with reduced hospital stay and mortality [11]. Antithrombotic therapy is also necessary post-TAVI to prevent thrombosis in the artificial valve. By performing TAVI and femoral surgery early in one session, antithrombotic therapy can be started promptly, potentially reducing both hemorrhagic and thrombotic complications.

There are several considerations to performing TAVI and femoral surgery in a single session. At our hospital, TAVI is only performed on designated days, which led to a 7-day wait between admission and surgery. Extended waiting periods may worsen outcomes for patients requiring femoral surgery [12, 13]. Additionally, bleeding and pain from the fracture can disrupt the myocardial oxygen supply–demand balance, increasing the risk of myocardial ischemia. In the future, it will be necessary to establish a system that enables emergency TAVI.

This was our first case of performing TAVI and non-cardiac surgery consecutively in a hybrid operating room. To perform femoral surgery following TAVI, the patient had to be transferred from the TAVI table to the traction table. Since the base of the TAVI table remains in the room, the positions of the traction table and surgical instruments were decided to avoid any interference with the base. Additionally, since the patient would be moved multiple times between tables, it was necessary to confirm the transfer routes. A simulation by multiple professionals was led by the anesthesiologists to review these points in advance, enabling the two consecutive surgeries to be performed smoothly.

When providing continuous anesthesia management for non-cardiac surgery following TAVI, attention must be paid to post-TAVI complications. It has been reported that complete AVB occurs in about 3%, and left bundle branch block in 40% of cases after TAVI with SAPIEN 3 [14]. In this case, although complete AVB did not occur, first-degree AVB and progressive bradycardia were observed during the osteosynthesis. Backup pacing by the temporary pacemaker was activated, and hemodynamics were maintained. Strict electrocardiogram monitoring and pacemaker management are important after TAVI.

Other major complication after TAVI is stroke. If stroke occur during TAVI, there may be a decrease in bispectral index (BIS) and cerebral regional oxygen saturation (rSO_2_), which could persist [15]. Although there was no significant drop in BIS in this case, rSO_2_ monitoring may have been necessary to detect early signs of reduced oxygen supply to the brain. Remimazolam was used for anesthesia, and the patient recovered quickly from anesthesia by administering flumazenil, allowing for confirmation of no stroke symptoms. However, performing both TAVI and femoral surgery simultaneously may extend the operation time, which could delay the detection of stroke and worsen the condition, so careful attention is needed.

## Conclusions

We performed TAVI and femoral osteosynthesis simultaneously on a patient with a hip fracture complicated by severe AS. Careful preparation and perioperative management led to a favorable outcome.

## Data Availability

Data sharing is not applicable to this article as no datasets were generated or analyzed during the current study.

## Abbreviations

AS: Aortic stenosis
TAVI: Transcatheter aortic valve implantation
Hb: Hemoglobin
AVB: Atrioventricular block
BAV: Balloon aortic valvuloplasty
BIS: Bispectral index
rSO_2_: Regional oxygen saturation
